# Anorectal function after transanal natural orifice specimen extraction for colorectal cancer: a systematic review and meta-analysis

**DOI:** 10.1007/s00423-026-04184-2

**Published:** 2026-08-05

**Authors:** Megan Wern Ee Chua, Ashley Shi Hui Lee, Prasad Palanisamy, Ian Jun Yan Wee, Isaac Seow-En

**Affiliations:** 1 Department of Colorectal Surgery, Singapore General Hospital, Outram Road, Singapore, 169608 Singapore; 2 Internal Medicine Residency, Singapore General Hospital, Singapore, 169608 Singapore

**Keywords:** Natural orifice specimen extraction (NOSE), Colorectal surgery, Transanal, Anorectal function, Transabdominal specimen extraction (TASE)

## Abstract

**Purpose:**

Natural orifice specimen extraction (NOSE) eliminates the abdominal extraction incision after minimally invasive (MIS) colorectal resection and offers favourable short-term recovery, but transanal extraction raises concerns regarding sphincter stretch and anorectal dysfunction. This review evaluated anorectal functional outcomes after transanal NOSE compared with transabdominal specimen extraction (TASE) in MIS colorectal cancer surgery.

**Methods:**

MEDLINE and EMBASE were searched from inception to August 2024 for comparative studies reporting postoperative anorectal or pelvic floor functional outcomes following transanal NOSE versus TASE. The primary outcome was anorectal continence at the postoperative interval closest to 6 months.

**Results:**

Ten retrospective cohort studies were included, of which seven (1362 patients) contributed quantitative data for meta-analysis. Three studies contributed to the pooled Wexner incontinence score analysis and four to the pooled Pelvic Floor Distress Inventory short form (PFDI-20) analysis. At the interval closest to 6 months, Wexner scores were numerically higher after transanal NOSE than TASE, but the difference was small and non-significant (MD 0.17, 95% CI − 0.01 to 0.35; *p* = 0.07). PFDI-20 scores did not differ between groups (MD − 0.02, 95% CI − 0.21 to 0.18; *p* = 0.86). Both estimates were robust in leave-one-out and overlap-adjusted analyses excluding potentially overlapping Harbin Medical University cohorts. Early differences appeared to diminish over time, with no clear separation beyond 12 months.

**Conclusion:**

Transanal NOSE was associated with a small, non-significant trend toward higher early Wexner scores after MIS colorectal cancer surgery, while broader pelvic floor symptom burden appeared comparable to TASE. The difference was of uncertain clinical significance, was robust to sensitivity analyses, and longer-term data suggested no persistent functional disadvantage.

**Supplementary Information:**

The online version contains supplementary material available at https://doi.org/10.1007/s00423-026-04184-2.

## Introduction

Minimally invasive colorectal surgery has substantially reduced access-related morbidity compared with open surgery; however, most procedures still require a mini-laparotomy for specimen extraction. This extraction incision remains an important contributor to postoperative pain, wound complications, and incisional hernia, sustaining interest in further minimising abdominal wall trauma.

Natural orifice specimen extraction (NOSE) surgery is one such strategy, allowing intracorporeal resection and reconstruction with specimen retrieval through a natural orifice, most commonly the anus or vagina, thereby avoiding an abdominal extraction incision. Accumulating evidence from comparative studies and meta-analyses suggests that NOSE is associated with reduced postoperative pain, faster recovery, and lower wound-related morbidity, while maintaining comparable oncological outcomes in appropriately selected patients [1–5].

Despite these advantages, transanal NOSE raises an important functional concern. Specimen extraction through the anal canal may expose the sphincter complex and pelvic floor to traction, distension, or local trauma, particularly in the setting of bulkier specimens or low rectal anastomoses. Any resulting impairment in anorectal function may offset the short-term benefits of avoiding an abdominal incision, especially given the established association between postoperative bowel dysfunction and health-related quality of life after sphincter-preserving colorectal surgery [6–7].

To date, anorectal functional outcomes after NOSE have been reported inconsistently across individual cohort studies, often as secondary endpoints within broader perioperative or oncological analyses. Existing systematic reviews have largely focused on short-term recovery and oncological outcomes, with limited synthesis of anorectal functional outcomes using validated instruments [1–5]. Consequently, the impact of transanal specimen extraction on continence and pelvic floor function remains uncertain.

In this systematic review and meta-analysis, we aimed to compare postoperative anorectal function between transanal NOSE and conventional transabdominal specimen extraction (TASE) following minimally invasive colorectal cancer surgery. By focusing on commonly used continence and pelvic floor assessment tools, including the Wexner incontinence score [8, 9] and the Pelvic Floor Distress Inventory short form (PFDI-20) [10], and by anchoring comparisons at a clinically relevant mid-term timepoint, we sought to clarify the functional safety profile of transanal NOSE and inform patient selection and counselling.

## Materials and methods

### Study design and reporting

This systematic review and meta-analysis was conducted in accordance with the Cochrane Handbook for Systematic Reviews of Interventions and reported in line with the Preferred Reporting Items for Systematic Reviews and Meta-Analyses (PRISMA) 2020 statement [11, 12]. The study was prospectively registered in the PROSPERO database (CRD42024539219) and conducted in accordance with the pre-specified protocol, with no major deviations.

### Literature search strategy

A systematic search of MEDLINE (via PubMed) and EMBASE was performed from database inception to August 2024. The search strategy combined controlled vocabulary (MeSH and Emtree terms) and free-text keywords relating to natural orifice specimen extraction, transanal extraction, colorectal cancer, and anorectal or pelvic floor function. Only studies published in English involving human participants were included. The full search strategy is provided in Supplementary Table S1.

Reference lists of included studies and relevant systematic reviews were manually screened to identify additional eligible studies.

### Eligibility criteria

Studies were eligible if they met the following criteria:

1. Included adult patients undergoing minimally invasive (laparoscopic or robotic) colorectal cancer resection;
2. Compared transanal natural orifice specimen extraction (NOSE) with conventional transabdominal specimen extraction (TASE);
3. Reported postoperative anorectal or pelvic floor functional outcomes using validated instruments (e.g. Wexner incontinence score or PFDI-20);
4. Included a comparator group.

Randomised controlled trials, prospective cohort studies, and retrospective cohort studies were eligible. Studies were excluded if they were non-comparative, conference abstracts, editorials, reviews, animal studies, or did not report extractable functional outcome data. Studies involving exclusively transvaginal NOSE were excluded. For studies including both transanal and transvaginal extraction, only studies with separately extractable transanal-specific functional data were included in quantitative synthesis. Mixed-route studies without extractable transanal data were excluded from pooled analysis to minimise confounding arising from differing pelvic floor mechanics between extraction approaches.

### Study selection and data extraction

Two reviewers (MCWE and PP) independently screened titles and abstracts, followed by full-text assessment for eligibility. Disagreements were resolved by consensus, with arbitration by a senior author (ISE) where required.

Data were extracted using a standardised, pre-piloted form. Extracted variables included study characteristics, patient demographics, tumour characteristics, treatment details, and functional outcomes, including the type of instrument used, follow-up timepoints, and reported scores.

### Outcomes

The primary outcome was postoperative anorectal continence at the follow-up interval closest to 6 months, assessed using the Wexner incontinence score [8]. The Wexner score is a five-item scale (range 0–20) evaluating incontinence to solid stool, liquid stool, gas, pad use, and lifestyle alteration, with higher scores indicating worse continence-related symptoms.

Secondary outcomes included broader pelvic floor symptom burden assessed using the Pelvic Floor Distress Inventory short form (PFDI-20) [10], as well as other reported anorectal functional measures. The PFDI-20 is a validated 20-item questionnaire assessing pelvic floor symptom burden, with higher scores indicating greater symptom severity.

Additional categorical continence measures reported by individual studies included Williams’ classification [13] and Kirwan grade [14].

Where multiple postoperative timepoints were available, data from the follow-up interval closest to 6 months were used for the primary analysis. The 6-month timepoint was selected to reflect a clinically relevant intermediate postoperative interval beyond the immediate postoperative recovery phase.

### Risk of bias assessment

Risk of bias for non-randomised studies was assessed independently by two reviewers using a modified Newcastle–Ottawa Scale (NOS) [15]. Studies were categorised as low (0–3), moderate (4–6), or high quality (7). Risk of bias was considered in the qualitative interpretation of results, and no studies were excluded based on NOS score. Discrepancies were resolved by consensus.

### Statistical analysis

Meta-analysis was performed using Review Manager (RevMan) using the inverse-variance method for continuous outcomes. Continuous outcomes were pooled using mean differences (MDs) with 95% confidence intervals (CIs), as all included studies reported outcomes using the same validated scales. Only directly reported means and standard deviations were pooled for quantitative synthesis.

Random-effects models were used for all analyses to account for anticipated clinical and methodological heterogeneity, with between-study variance estimated using the restricted maximum likelihood method. For the overlap-adjusted sensitivity analyses, in which only two studies remained per outcome after exclusion of potentially overlapping cohorts, a fixed-effect model was used, as estimation of between-study variance is unreliable and random-effects pooling offers no advantage with so few studies and low observed heterogeneity.

Statistical heterogeneity was assessed using the I² statistic, with values of < 25% considered low, 25–50% moderate, and > 50% substantial heterogeneity.

Leave-one-out sensitivity analyses were performed by sequential exclusion of individual studies to assess the robustness of pooled estimates. To address potential patient overlap among studies originating from the Affiliated Hospital of Harbin Medical University, which shared overlapping recruitment periods, an overlap-adjusted sensitivity analysis was additionally performed for each outcome. Within the Harbin cluster, a single representative cohort was retained for each outcome and the remaining potentially overlapping Harbin studies were excluded, and pooled estimates were recalculated. The representative cohort was the Harbin study with the most complete and appropriate functional data and the broadest, most representative inclusion criteria among those contributing to that outcome; because the studies contributing to the Wexner and PFDI-20 analyses differed, the retained representative cohort also differed between outcomes. Results of the leave-one-out and overlap-adjusted analyses are presented in Supplementary Figures S1–S3 and Supplementary Tables S3–S4.

Due to the small number of studies included in each analysis (< 10), formal assessment of publication bias using funnel plots was not performed [16]. Separate subgroup analyses by tumour location (colon versus rectum) were not feasible due to lack of stratified reporting in the included studies.

## Results

### Study selection

**Fig. 1:**
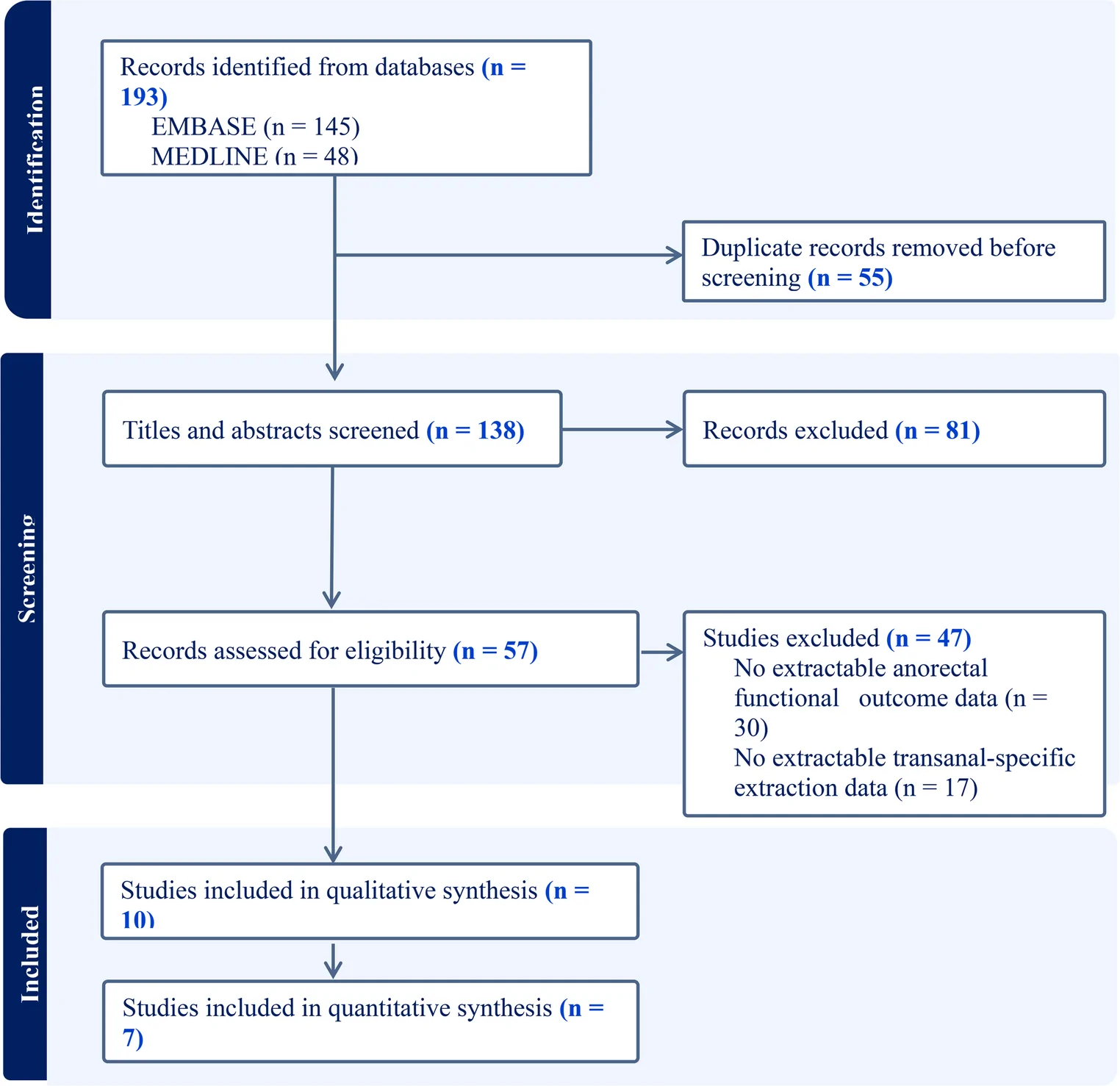
PRISMA 2020 flow diagram of study selection for the systematic review and meta-analysis. Records identified through MEDLINE and EMBASE database searching underwent sequential screening, eligibility assessment, and inclusion for qualitative synthesis and quantitative meta-analysis of postoperative anorectal functional outcomes following transanal natural orifice specimen extraction (NOSE) versus conventional transabdominal specimen extraction (TASE) in minimally invasive colorectal cancer surgery

The literature search identified 193 records, comprising 48 from MEDLINE and 145 from EMBASE. After removal of 55 duplicates, 138 records underwent title and abstract screening, of which 57 articles proceeded to full-text review. Forty-seven studies were excluded following full-text assessment, primarily due to absence of extractable functional outcome data or lack of transanal-specific extraction data. Ten studies were included in the qualitative synthesis, and seven contributed extractable quantitative data for meta-analysis. The study selection process is summarised in Fig. 1.

### Methodological quality and risk of bias

Methodological quality was moderate-to-high according to the modified Newcastle–Ottawa Scale assessment. Four studies achieved the maximum score of 7, five studies scored 6, and one study scored 5. Detailed risk-of-bias assessment is presented in Supplementary Table S2.

All included studies were retrospective cohort studies, and most were single-centre studies from China. Several studies originated from overlapping institutions and recruitment periods, particularly from the Affiliated Hospital of Harbin Medical University, raising the possibility of partial patient overlap between cohorts.

### Characteristics of included studies

Ten studies published between 2021 and 2024 were included [17–26]. Sample sizes ranged from 76 to 372 patients.

All studies compared transanal NOSE with conventional transabdominal specimen extraction following minimally invasive colorectal cancer surgery. Most cohorts included predominantly rectal and sigmoid tumours, although two studies additionally included selected left-sided colonic lesions [17, 26]. Most studies included relatively small tumours, generally around or below 5 cm in diameter, and the study populations were largely composed of patients with non-metastatic stage II–III disease.

Functional outcomes were most commonly assessed using the Wexner incontinence score (nine studies) and PFDI-20 (five studies). Additional categorical continence assessments using Williams’ classification and Kirwan grade were reported in one study [22].

Characteristics of the included studies are summarised in Tables 1 and 2.

**Table 1:** Baseline characteristics of studies included in the systematic review and meta-analysis

Author	Country	Setting	Study design	Recruitment period	Sample size (NOSE/TASE)	Matching method	Mean age, years (NOSE/TASE)	Male sex, *n* (%) (NOSE/TASE)	BMI, kg/m² (NOSE/TASE)
Tang et al. [17]	China	Single-centre, Second Affiliated Hospital of Harbin Medical University	Retrospective cohort	2013–2017	186 / 186	Propensity-score matched	59.0 ± 10.4 / 59.9 ± 11.3	94 (50.5) / 92 (49.5)	22.9 ± 2.8 / 22.8 ± 3.2
Zhong et al. [18]	China	Single-centre, Affiliated Hospital of Harbin Medical University	Retrospective cohort	2013–2017	38 / 38	Matched retrospective cohort	57.8 ± 10.3 / 60.9 ± 9.9	21 (55.3) / 22 (57.9)	22.7 ± 2.5 / 22.4 ± 2.8
Zhu et al. [19]	China	Single-centre, Affiliated Hospital of Harbin Medical University	Retrospective cohort	2013–2015	59 / 59	Matched retrospective cohort	59.6 ± 12.1 / 59.8 ± 10.4	29 (49.2) / 36 (61.0)	23.3 ± 2.7 / 23.0 ± 2.9
Li et al. [20]	China	Single-centre, Affiliated Hospital of Harbin Medical University	Retrospective cohort	2013–2017	186 / 106	Propensity-score matched	59.5 ± 11.5 / 59.9 ± 11.1	104 (55.9) / 61 (57.5)	22.1 ± 2.9 / 22.9 ± 3.1
Zhang et al. [21]	China	Single-centre, Affiliated Hospital of Harbin Medical University	Retrospective cohort	2013–2017	39 / 39	Propensity-score matched	72.7 ± 5.8 / 71.4 ± 5.1	19 (48.7) / 20 (51.3)	21.5 ± 3.2 / 22.9 ± 3.0
Zhao et al. [22]	China	Single-centre, Affiliated Hospital of Chengde Medical College	Retrospective cohort	2014–2020	60 / 60	Unmatched retrospective cohort	61.1 ± 10.2 / 59.6 ± 10.6	29 (48.3) / 38 (63.3)	24.1 ± 3.2 / 25.1 ± 3.9
Zhang et al. [23]	China	Single-centre, Beijing Shijitan Hospital	Retrospective cohort	2012–2016	69 / 69	Matched retrospective cohort	54.4 ± 11.6 / 55.3 ± 11.5	37 (53.6) / 37 (53.6)	23.3 ± 3.3 / 23.2 ± 3.3
Ju et al. [24]	China	Single-centre, Affiliated Hospital of Nanchang University	Retrospective cohort	2017–2020	138 / 144	Propensity-score matched	60 ± 8 / 63 ± 7	76 (55.1) / 100 (69.4)	22.4 ± 2.5 / 22.7 ± 2.8
Muhammad et al. [25]	China	Multicentre study	Retrospective cohort	2013–2017	44 / 92	Matched retrospective cohort	58.9 ± 11.6 / 58.3 ± 10.5	21 (47.7) / 54 (58.7)	22.8 ± 2.9 / 22.7 ± 3.0
Xu et al. [26]	China	Single-centre, Zhongshan Hospital Xiamen University	Retrospective cohort	2014–2021	132 / 132	Propensity-score matched	61.8 ± 10.3 / 60.6 ± 12.6	77 (58.3) / 75 (56.8)	22.8 ± 3.6 / 22.2 ± 2.5

*BMI* body mass index, *NOSE* natural orifice specimen extraction, *TASE* transabdominal specimen extraction

Values are presented as NOSE/TASE unless otherwise specified. Continuous variables are reported as mean ± standard deviation. Baseline data were extracted preferentially from propensity score–matched cohorts where available

**Table 2:** Tumour characteristics, extraction approaches, and anorectal functional outcome assessment among included studies

Author	Tumour location	Tumour size criteria / reported size	Extraction route	Functional assessment tools	Functional assessment timepoints	Key functional findings	Contribution to pooled analysis
Tang et al. [17]	Colorectal cancer (rectum predominant)	Tumour diameter ≤ 5 cm	Transanal and transvaginal	Wexner score, PFDI-20	PFDI-20 at 3 months; Wexner at 6 months	No significant difference in postoperative PFDI-20 or Wexner scores between groups	PFDI-20 meta-analysis
Zhong et al. [18]	Middle rectal cancer	Not clearly reported	Transanal	Wexner score	6 and 12 months	Comparable Wexner scores between groups	Wexner meta-analysis
Zhu et al. [19]	Low rectal cancer	Not clearly reported	Transanal	PFDI-20	3 months	No significant difference in postoperative PFDI-20 scores	PFDI-20 meta-analysis
Li et al. [20]	Rectal cancer	Not clearly reported	Transanal	PFDI-20	Closest to 6 months	No significant difference in postoperative PFDI-20 scores between groups	PFDI-20 meta-analysis
Zhang et al. [21]	Colorectal cancer in elderly patients	Not clearly reported	Transanal	Not specifically reported	Not reported	Functional outcomes not quantitatively reported	Qualitative synthesis only
Zhao et al. [22]	Rectal cancer	Mean tumour size approximately 3–4 cm	Transanal	Wexner score, Williams classification, Kirwan grade	6 months	Comparable continence outcomes between groups	Qualitative synthesis only
Zhang et al. [23]	Sigmoid colon and rectal cancer	Mean tumour size approximately 3 cm	Transanal	Wexner score	1 month, 3 months, > 5 years	Higher early postoperative Wexner scores following NOSE, with no significant long-term difference	Wexner meta-analysis
Ju et al. [24]	Colorectal cancer	Not clearly reported	Transanal	Wexner score, PFDI-20	PFDI-20 at 3 months; Wexner at 6 months	No significant difference in postoperative PFDI-20 or Wexner scores	PFDI-20 meta-analysis
Muhammad et al. [25]	Mid-rectal cancer	Tumour diameter ≤ 5 cm	Transanal	PFDI-20	3 months	No significant difference in postoperative PFDI-20 scores	Qualitative synthesis only
Xu et al. [26]	Left-sided colorectal cancer (descending/sigmoid/upper rectum)	Tumour diameter ≤ 4.5 cm	Transanal using Cai tube	Wexner score	6 and 12 months	Comparable Wexner scores between groups	Wexner meta-analysis

*NOSE* natural orifice specimen extraction, *PFDI-20* Pelvic Floor Distress Inventory short form, *TASE* transabdominal specimen extraction

Functional assessment timepoints refer to postoperative follow-up intervals reported in the original studies. Tumour size criteria reflect either study eligibility criteria or reported mean tumour size where available. Extraction route refers specifically to the NOSE cohort

### Quantitative synthesis

Seven studies comprising 1362 patients contributed extractable quantitative data for meta-analysis. Three studies contributed to pooled Wexner incontinence score analysis [18, 23, 26], while four studies contributed to pooled PFDI-20 analysis [17, 19, 20, 24]. On re-verification of the extracted data during revision, Ju et al. [24] were found not to report extractable quantitative Wexner data and were therefore removed from the pooled Wexner analysis; Ju et al. [24] continued to contribute to the pooled PFDI-20 analysis. The contribution of each study to the qualitative and quantitative syntheses is summarised in Table 2.

### Postoperative anorectal function at the follow-up interval closest to 6 months

**Fig. 2:**
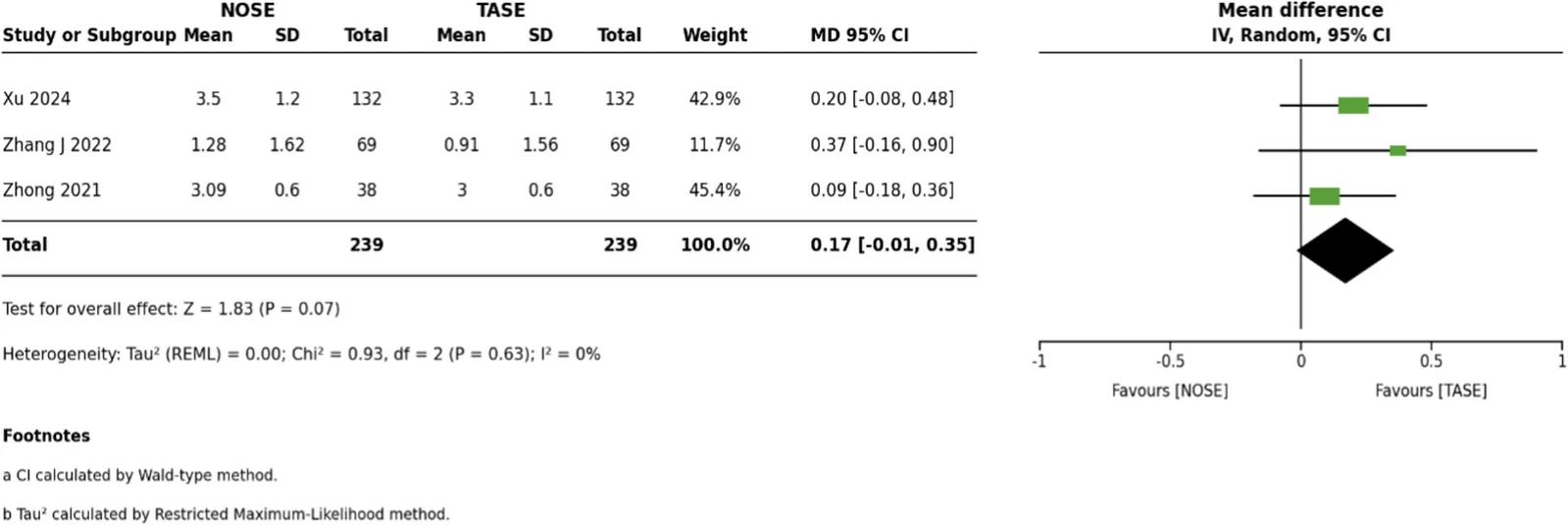
Forest plot comparing postoperative Wexner incontinence scores between transanal NOSE and transabdominal specimen extraction (TASE) at the postoperative interval closest to 6 months. Positive mean differences indicate higher Wexner scores following transanal NOSE

Most included studies reported comparable anorectal functional outcomes between NOSE and TASE at the postoperative follow-up interval closest to 6 months. Pooled analysis of three studies demonstrated a small, numerically higher Wexner incontinence score in the transanal NOSE group compared with TASE that did not reach statistical significance (MD 0.17, 95% CI − 0.01 to 0.35; *p* = 0.07; I²=0%) (Fig. 2), indicating at most a small trend toward worse continence-related symptoms following transanal extraction. The direction and magnitude of this effect were consistent across leave-one-out sensitivity analyses (pooled MD ranging from 0.14 to 0.20; all *p* > 0.05; Supplementary Figure S1) and in the overlap-adjusted analysis excluding potentially overlapping Harbin Medical University cohorts (MD 0.24, 95% CI − 0.01 to 0.48; *p* = 0.06; Supplementary Figure S3).

**Fig. 3:**
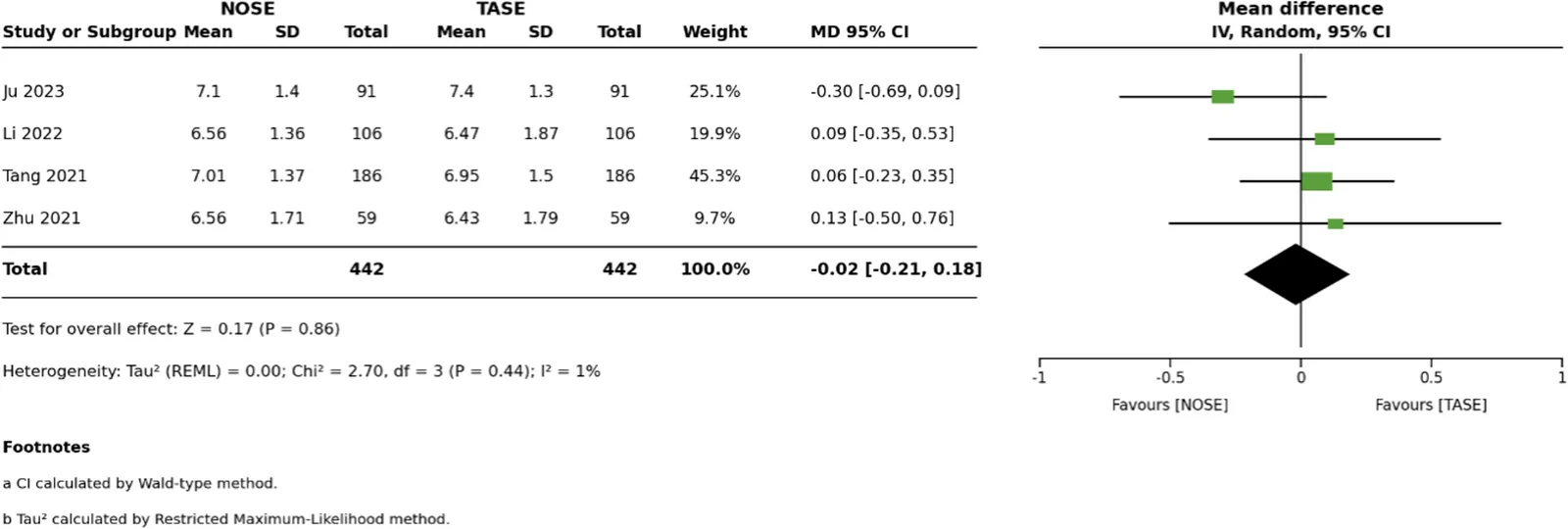
Forest plot comparing postoperative PFDI-20 scores between transanal natural orifice specimen extraction (NOSE) and conventional transabdominal specimen extraction (TASE) at the follow-up interval closest to 6 months. Pooled mean differences were calculated using a random-effects model. Higher scores indicate greater pelvic floor symptom burden

In contrast, pooled analysis of PFDI-20 scores demonstrated no statistically significant difference between groups (MD − 0.02, 95% CI − 0.21 to 0.18; *p* = 0.86; I²=1%) (Fig. 3), suggesting comparable broader pelvic floor symptom burden between transanal NOSE and TASE.

Statistical heterogeneity was low in both pooled analyses. Leave-one-out sensitivity analyses demonstrated consistent findings for both outcomes, with no individual study materially altering the pooled estimate or its statistical significance (Supplementary Figures S1 and S2; Supplementary Tables S3 and S4). Overlap-adjusted analyses were performed for both outcomes by retaining a single representative cohort from the Affiliated Hospital of Harbin Medical University and excluding the remaining potentially overlapping Harbin studies. Because the contributing studies differed between outcomes, the studies retained also differed. For the PFDI-20 analysis, Li et al. [20] was retained as the representative Harbin cohort, and the overlapping Harbin studies (Tang [17] and Zhu [19]) were excluded, leaving Li [20] and the non-Harbin cohort Ju [24]. For the Wexner analysis, in which Li et al. did not contribute extractable data, the only Harbin-cluster Wexner contributor (Zhong [18]) was excluded, leaving the non-overlapping studies with extractable Wexner data (Zhang J [23] and Xu [26]). In both analyses the pooled estimates remained consistent in direction and magnitude with the primary analyses, for both Wexner (MD 0.24, 95% CI − 0.01 to 0.48; *p* = 0.06; I²=0%) and PFDI-20 scores (MD − 0.13, 95% CI − 0.42 to 0.17; *p* = 0.39; I²=40%), albeit with reduced precision owing to the smaller number of pooled studies (Supplementary Figure S3).

### Early postoperative anorectal function

Three studies reported functional outcomes within the early postoperative period (1–3 months) [19, 23, 25].

Zhang et al. reported higher Wexner scores in the NOSE group at both 1 month and 3 months postoperatively compared with TASE, with scores in both groups remaining elevated relative to baseline [23]. These findings suggest transient early postoperative deterioration in anorectal function following surgery, with a greater degree of impairment observed following transanal extraction.

In contrast, studies evaluating PFDI-20 did not demonstrate significant differences between groups during early follow-up [19, 25].

### Longer-term anorectal function

Two studies reported longer-term functional outcomes beyond 12 months [23, 26]. Both studies demonstrated improvement in Wexner scores over time, with no statistically significant differences between NOSE and TASE groups at later follow-up intervals. These observations are based on cross-sectional comparisons reported at differing postoperative timepoints within retrospective cohorts rather than on prospective, paired longitudinal assessment of the same patients over time, and should therefore be interpreted cautiously as suggestive rather than confirmatory of a trajectory of recovery.

Additional categorical continence assessments using Williams’ classification and Kirwan grade similarly demonstrated comparable long-term continence outcomes between groups [22].

## Discussion

The present systematic review and meta-analysis evaluated anorectal functional outcomes following transanal natural orifice specimen extraction (NOSE) compared with conventional transabdominal specimen extraction (TASE) in minimally invasive colorectal cancer surgery. Pooled analysis demonstrated a small, numerically higher Wexner incontinence score following transanal NOSE at follow-up closest to 6 months that did not reach statistical significance (MD 0.17 on a 20-point scale; *p* = 0.07), and the absolute difference was of uncertain clinical significance. In contrast, broader pelvic floor symptom burden assessed using PFDI-20 did not differ between groups. These findings were robust to leave-one-out and overlap-adjusted sensitivity analyses that accounted for potential patient overlap among studies originating from the Affiliated Hospital of Harbin Medical University. Overall, these findings suggest that transanal NOSE may be associated with at most a modest, non-significant trend toward early continence-related symptoms, without clear evidence of persistent anorectal dysfunction.

The short-term perioperative benefits of NOSE are increasingly established. Multiple meta-analyses have demonstrated reductions in postoperative pain, wound morbidity, and recovery time while maintaining comparable oncological outcomes in appropriately selected patients [1–5]. However, anorectal functional outcomes remain comparatively underexplored despite their importance in sphincter-preserving colorectal surgery.

The small difference in Wexner score observed in the present analysis should be interpreted cautiously. The pooled difference did not reach statistical significance and, even if a true difference exists, its absolute magnitude was minimal and unlikely to represent clinically meaningful impairment from a patient perspective. Although a formally established minimal clinically important difference (MCID) for the Wexner score is lacking, differences of this order (well below one point on a 0–20 scale) fall far short of the several-point changes generally regarded as clinically relevant for continence scores, reinforcing the limited clinical importance of the observed difference. Importantly, studies with serial follow-up demonstrated improvement over time, with no clear long-term separation between groups beyond 12 months [23, 26]. This pattern suggests that any postoperative dysfunction may be transient rather than reflective of permanent sphincter injury. These observations derive from cross-sectional comparisons at differing timepoints within retrospective cohorts rather than from prospective paired longitudinal assessment, and should therefore be regarded as hypothesis-generating.

Several mechanisms may contribute to transient anorectal dysfunction following transanal specimen extraction. Delivery of the specimen through the anal canal may result in temporary sphincter stretch, tissue oedema, or traction-related pelvic floor stress, particularly in technically challenging extraction cases or with bulkier specimens. Functional outcomes may additionally be influenced by specimen bulk, mesenteric thickness, extraction force, use of protective extraction devices, and the degree of anal dilatation required. Xu et al., for example, described use of a Cai tube-assisted extraction technique [26], which may theoretically reduce direct sphincter trauma during extraction. However, extraction techniques were inconsistently reported across studies, limiting further analysis. More broadly, postoperative anorectal dysfunction following rectal surgery is multifactorial and influenced by pelvic dissection, rectal reservoir loss, autonomic nerve injury, anastomotic height, and neoadjuvant treatment [27]. Specimen extraction itself therefore likely represents only one component within a broader functional pathway.

Contemporary NOSE practice already incorporates substantial patient selection. International consensus statements recommend careful consideration of tumour size, body habitus, pelvic anatomy, and specimen characteristics when selecting patients for transanal extraction [28, 29]. Most studies included relatively small tumours, predominantly rectal or sigmoid lesions, thereby limiting extrapolation to bulkier T3/T4 lesions or obese patients requiring more technically demanding extraction. Feasibility studies have additionally demonstrated that anatomical and tumour-related factors strongly influence successful NOSE implementation [30]. Consequently, the modest functional differences observed in the present analysis may partly reflect appropriate case selection within experienced centres.

The choice of functional instruments also warrants consideration. The Wexner score captures only the incontinence dimension of anorectal dysfunction and does not assess urgency, clustering, or incomplete evacuation. The PFDI-20 was originally developed and validated in women, and its psychometric validity in men has not been established; as approximately half of the pooled populations were male, and none of the studies reported sex-specific administration or scoring, the pooled PFDI-20 estimates should be interpreted with this caveat. These are limitations of the primary literature rather than of the present synthesis, but they constrain the strength of inference, and future studies would benefit from validated, sex-appropriate, procedure-specific measures.

A further limitation is the absence of Low Anterior Resection Syndrome (LARS) score data. The LARS score is now the most widely validated instrument for the bowel symptoms—urgency, clustering, frequency, and incomplete evacuation, in addition to incontinence—that follow sphincter-preserving rectal surgery, and is arguably the more clinically relevant measure in this setting. None of the included studies employed it. The present findings, resting on the Wexner score and PFDI-20, therefore cannot address the full spectrum of post-resection functional impairment, and this gap should be a priority for future prospective studies.

Several limitations should be acknowledged. All included studies were retrospective cohorts, susceptible to selection bias and residual confounding; where propensity-score matching was used, it was largely confined to demographic and tumour variables and did not account for factors plausibly related to anorectal function (anastomotic height, neoadjuvant therapy, specimen size, prior pelvic surgery). Most studies were single-centre Chinese cohorts, limiting generalisability; differences in body habitus and pelvic anatomy between Asian and Western populations [31] may further influence extraction mechanics and the applicability of these findings. Several studies originated from overlapping institutions and recruitment periods, particularly the Affiliated Hospital of Harbin Medical University [17–21], raising the possibility of patient overlap that, if present, would violate the independence assumption of meta-analytic pooling and inflate apparent precision. We could not obtain individual patient data to quantify this; however, the overlap-adjusted and leave-one-out analyses preserved the direction and magnitude of the pooled estimates, indicating that the principal findings are unlikely to be artefacts of overlap, albeit with reduced precision. Functional outcome reporting was heterogeneous, limiting quantitative synthesis to a subset of studies. The search was restricted to MEDLINE and EMBASE and to English-language publications, current to August 2024; CENTRAL and Web of Science were not searched, and relevant Chinese-language data may have been missed. Finally, the small number of studies (fewer than ten per outcome) precluded formal funnel-plot assessment of publication bias, which nonetheless remains a qualitative concern given the tendency for small positive single-centre studies to be preferentially published.

Despite these limitations, this study represents, to our knowledge, among the first systematic reviews and meta-analyses specifically focused on anorectal functional outcomes following transanal NOSE in minimally invasive colorectal cancer surgery. By focusing on established continence and pelvic floor outcome measures at clinically relevant postoperative timepoints, this study provides a more focused assessment of the functional safety profile of transanal extraction than prior NOSE meta-analyses centred primarily on perioperative or oncological outcomes.

Further prospective multicentre studies using standardised functional assessment protocols and longer follow-up are required to better define the clinical relevance and trajectory of postoperative anorectal dysfunction following transanal NOSE. Future studies should additionally evaluate patient-reported quality-of-life outcomes and explore technical, anatomical, and specimen-related factors associated with increased functional risk.

## Conclusion

Transanal NOSE was associated with at most a small, statistically non-significant trend toward higher continence-related symptoms at approximately 6 months following minimally invasive colorectal cancer surgery, while broader pelvic floor symptom burden appeared comparable to conventional transabdominal extraction. The magnitude of any difference in Wexner scores was small and of uncertain clinical significance, the pooled estimates were robust to leave-one-out and overlap-adjusted sensitivity analyses, and available longer-term data suggested no clear persistent functional disadvantage. These findings are constrained by the retrospective nature and potential cohort overlap of the included studies, the absence of LARS score data, and uncertainty regarding the validity of the PFDI-20 in mixed-sex populations. Further prospective multicentre studies using standardised, validated, and procedure-specific functional assessment instruments, including the LARS score, are required. 

## Supplementary Information

Below is the link to the electronic supplementary material.

Supplementary Material 1 (PDF 1.12 MB)

## Data Availability

The datasets analysed during the current study are available from the corresponding author upon reasonable request.

## Declarations

### **PROSPERO registration**

This systematic review and meta-analysis was prospectively registered in the PROSPERO database (CRD42024539219).
