# Supplementary material for: Anorectal function after transanal natural orifice specimen extraction for colorectal cancer: a systematic review and meta-analysis
Source: Langenbecks Arch Surg. 2026 Aug 5;411(1):303. doi: 10.1007/s00423-026-04184-2 (PMC13727840; doi:10.1007/s00423-026-04184-2)

Supplementary Material

Anorectal function after transanal natural orifice specimen extraction for colorectal cancer: a systematic review and meta-analysis

Langenbeck's Archives of Surgery

Corresponding author: Isaac Seow-En, Department of Colorectal Surgery, Singapore General Hospital, Outram Road, Singapore 169608. Email: isaac.seow.en@gmail.com

Contents

Supplementary Table S1. Search strategy for MEDLINE (via PubMed) and EMBASE
Supplementary Table S2. Modified Newcastle–Ottawa Scale assessment of included studies
Supplementary Table S3. Leave-one-out sensitivity analysis for the pooled Wexner incontinence score
Supplementary Table S4. Leave-one-out sensitivity analysis for the pooled PFDI-20 score
Supplementary Figure S1. Leave-one-out sensitivity analysis for the pooled Wexner incontinence score
Supplementary Figure S2. Leave-one-out sensitivity analysis for the pooled PFDI-20 score
Supplementary Figure S3. Overlap-adjusted pooled analysis excluding potentially overlapping Harbin cohorts

Supplementary Table S1. Search strategy for MEDLINE (via PubMed) and EMBASE

Search concept	Search terms
Natural orifice specimen extraction terms	“natural orifice specimen extraction” OR NOSE OR NOSES OR transanal specimen extraction OR transanal extraction
Colorectal cancer surgery terms	colorectal cancer OR rectal cancer OR colon cancer OR colorectal neoplasms OR colorectal surgery
Functional outcome terms	anorectal function OR bowel function OR continence OR incontinence OR fecal incontinence OR faecal incontinence OR pelvic floor OR pelvic floor dysfunction OR Wexner OR PFDI OR PFDI-20
Combined search strategy	(#1) AND (#2) AND (#3)

Searches were performed from database inception to 31 August 2024.

Limits applied:

- Human studies
- English language
- No publication year restriction

Representative full PubMed search syntax

(("natural orifice specimen extraction"[Title/Abstract]
OR NOSE[Title/Abstract]
OR NOSES[Title/Abstract]
OR "transanal specimen extraction"[Title/Abstract]
OR "transanal extraction"[Title/Abstract])
AND
("colorectal cancer"[Title/Abstract]
OR "rectal cancer"[Title/Abstract]
OR "colon cancer"[Title/Abstract]
OR "colorectal neoplasms"[MeSH Terms]

OR "colorectal surgery"[Title/Abstract])
AND
("anorectal function"[Title/Abstract]
OR "bowel function"[Title/Abstract]
OR continence[Title/Abstract]
OR incontinence[Title/Abstract]
OR "fecal incontinence"[Title/Abstract]
OR "faecal incontinence"[Title/Abstract]
OR "pelvic floor"[Title/Abstract]
OR "pelvic floor dysfunction"[Title/Abstract]
OR Wexner[Title/Abstract]
OR PFDI[Title/Abstract]
OR PFDI-20[Title/Abstract]))

EMBASE

A comparable search strategy adapted for EMBASE indexing terms and syntax was performed using the same core concepts and keywords.

Supplementary Table S2. Modified Newcastle–Ottawa Scale assessment of included studies

**Study ID**	Representativeness of exposed cohort (max 1)	Selection of non-exposed cohort (max 1)	Ascertainment of exposure (max 1)	Comparability of cohorts (max 2)	Outcome assessment (max 1)	Adequacy of follow up (max 1)	Total (out of 7)
Tang et al. [17]	★	★	★	★	★	★	★★★★★★ (6)
Zhong et al. [18]	★	★	★	★	★	★	★★★★★★ (6)
Zhu et al. [19]	★	★	★	★	★	★	★★★★★★ (6)
Li et al. [20]	★	★	★	★	★	★	★★★★★★ (6)
Zhang et al. [21]	★	★	★	★★	★	★	★★★★★★★ (7)
Zhao et al. [22]	★	★	★	★	★	-	★★★★★ (5)
Zhang et al. [23]	★	★	★	★	★	★	★★★★★★ (6)
Ju et al. [24]	★	★	★	★★	★	★	★★★★★★★ (7)
Muhammad et al. [25]	★	★	★	★★	★	★	★★★★★★★ (7)
Xu et al. [26]	★	★	★	★★	★	★	★★★★★★★ (7)

Studies scoring 6–7 were considered moderate-to-high quality, 4–5 moderate quality, and 0–3 low quality.
Comparability scores of 2 were awarded to studies employing propensity score matching or robust adjustment for major baseline confounders.

Supplementary Table S3. Leave-one-out sensitivity analysis for the pooled Wexner incontinence score

Sensitivity analysis	Studies included	Pooled MD	95% CI	p-value	I^2
Main Wexner analysis	Zhong 2021 + Zhang J 2022 + Xu 2024	0.17	−0.01 to 0.35	0.07	0%
Omit Zhong 2021	Zhang J 2022 + Xu 2024	0.20	−0.08 to 0.48	0.16	0%
Omit Zhang J 2022	Zhong 2021 + Xu 2024	0.14	−0.05 to 0.34	0.15	0%
Omit Xu 2024	Zhong 2021 + Zhang J 2022	0.15	−0.09 to 0.39	0.23	0%

MD, mean difference; CI, confidence interval. Pooled estimates were calculated using a random-effects model (inverse-variance method, restricted maximum-likelihood estimation of between-study variance). The main analysis included the three studies contributing extractable quantitative Wexner data (Zhong 2021, Zhang J 2022, and Xu 2024) after exclusion of Ju et al., which did not report extractable quantitative Wexner data. Sequential omission of each study did not materially alter the pooled estimate, which remained small and statistically non-significant throughout.

Supplementary Table S4. Leave-one-out sensitivity analysis for the pooled PFDI-20 score

Sensitivity analysis	Studies included	Pooled MD	95% CI	p-value	I^2
Main PFDI-20 analysis	Tang 2021 + Zhu 2021 + Li 2022 + Ju 2023	−0.02	−0.21 to 0.18	0.86	1%
Omit Tang 2021	Zhu 2021 + Li 2022 + Ju 2023	−0.07	−0.37 to 0.23	0.64	9%
Omit Zhu 2021	Tang 2021 + Li 2022 + Ju 2023	−0.04	−0.27 to 0.19	0.75	18%
Omit Li 2022	Tang 2021 + Zhu 2021 + Ju 2023	−0.05	−0.32 to 0.21	0.71	24%
Omit Ju 2023	Tang 2021 + Zhu 2021 + Li 2022	0.08	−0.15 to 0.30	0.51	0%

MD, mean difference; CI, confidence interval; PFDI-20, Pelvic Floor Distress Inventory short form. Pooled estimates were calculated using a random-effects model (inverse-variance method, restricted maximum-likelihood estimation of between-study variance). The main analysis

included the four studies contributing extractable quantitative PFDI-20 data (Tang 2021, Zhu 2021, Li 2022, and Ju 2023). Sequential omission of each study did not materially alter the pooled estimate, which remained small and statistically non-significant throughout.

Supplementary Figure S1. Leave-one-out sensitivity analysis for the pooled Wexner incontinence score. Forest plots show the pooled mean difference in Wexner score between transanal NOSE and TASE for (a) all three contributing studies and (b–d) after sequential omission of each study. Pooled estimates were calculated using a random-effects model. The pooled estimate remained small and statistically non-significant throughout.

(a) All studies (main Wexner analysis)

	NOSE			TASE				
**Study or Subgroup**	**Mean**	**SD**	**Total**	**Mean**	**SD**	**Total**	**Weight**	**MD 95% CI**
Xu 2024	3.5	1.2	132	3.3	1.1	132	42.9%	0.20 [-0.08, 0.48]
Zhang J 2022	1.28	1.62	69	0.91	1.56	69	11.7%	0.37 [-0.16, 0.90]
Zhong 2021	3.09	0.6	38	3	0.6	38	45.4%	0.09 [-0.18, 0.36]
**Total**			**239**			**239**	**100.0%**	**0.17 [-0.01, 0.35]**

Test for overall effect: Z = 1.83 (P = 0.07)

Heterogeneity: Tau² (REML) = 0.00; Chi² = 0.93, df = 2 (P = 0.63); I² = 0%

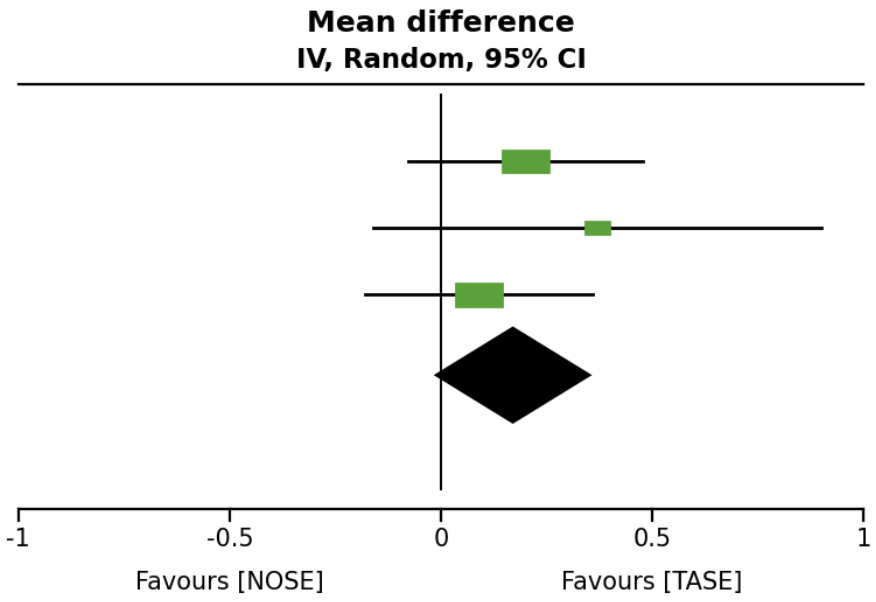

Footnotes

a CI calculated by Wald-type method.

b Tau² calculated by Restricted Maximum-Likelihood method.

(b) Omitting Zhong 2021

	NOSE			TASE				
**Study or Subgroup**	**Mean**	**SD**	**Total**	**Mean**	**SD**	**Total**	**Weight**	**MD 95% CI**
Xu 2024	3.5	1.2	132	3.3	1.1	132	78.5%	0.20 [-0.08, 0.48]
Zhang J 2022	1.28	1.62	69	0.91	1.56	69	21.5%	0.37 [-0.16, 0.90]
**Total**			**201**			**201**	**100.0%**	**0.20 [-0.08, 0.48]**

Test for overall effect: Z = 1.40 (P = 0.16)

Heterogeneity: Tau² (REML) = 0.00; Chi² = 0.31, df = 1 (P = 0.58); I² = 0%

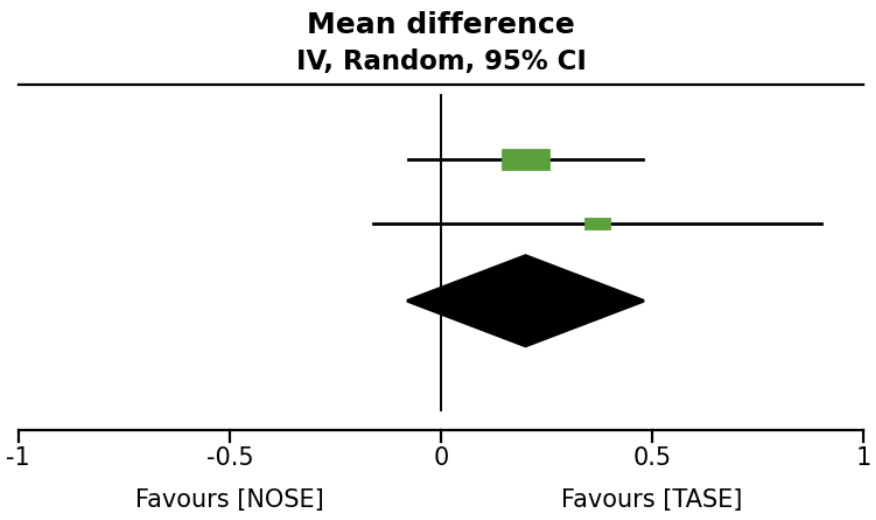

Footnotes

a CI calculated by Wald-type method.

b Tau² calculated by Restricted Maximum-Likelihood method.

(c) Omitting Zhang J 2022

	NOSE			TASE				
**Study or Subgroup**	**Mean**	**SD**	**Total**	**Mean**	**SD**	**Total**	**Weight**	**MD 95% CI**
Xu 2024	3.5	1.2	132	3.3	1.1	132	48.6%	0.20 [-0.08, 0.48]
Zhong 2021	3.09	0.6	38	3	0.6	38	51.4%	0.09 [-0.18, 0.36]
**Total**			**170**			**170**	**100.0%**	**0.14 [-0.05, 0.34]**

Test for overall effect: Z = 1.45 (P = 0.15)

Heterogeneity: Tau² (REML) = 0.00; Chi² = 0.31, df = 1 (P = 0.58); I² = 0%

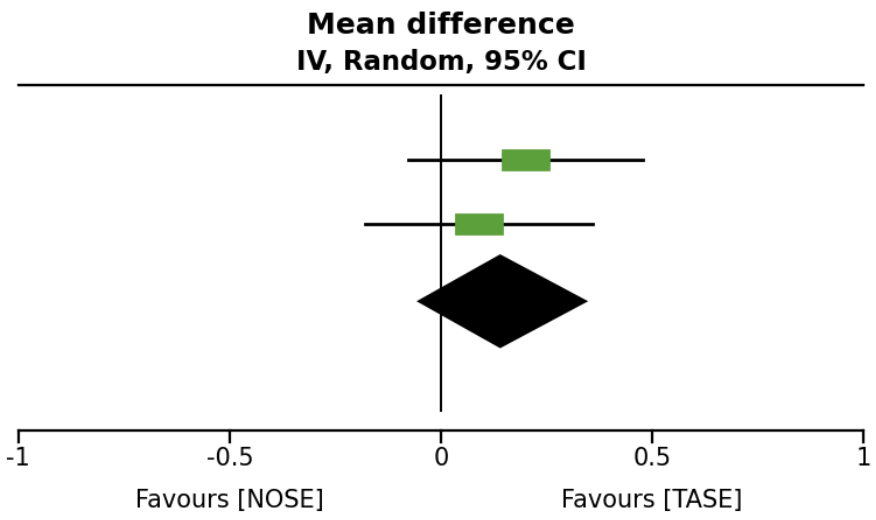

Footnotes

a CI calculated by Wald-type method.

b Tau² calculated by Restricted Maximum-Likelihood method.

(d) Omitting Xu 2024

	NOSE			TASE				
**Study or Subgroup**	**Mean**	**SD**	**Total**	**Mean**	**SD**	**Total**	**Weight**	**MD 95% CI**
Zhang J 2022	1.28	1.62	69	0.91	1.56	69	20.5%	0.37 [-0.16, 0.90]
Zhong 2021	3.09	0.6	38	3	0.6	38	79.5%	0.09 [-0.18, 0.36]
**Total**			**107**			**107**	**100.0%**	**0.15 [-0.09, 0.39]**

Test for overall effect: Z = 1.20 (P = 0.23)

Heterogeneity: Tau² (REML) = 0.00; Chi² = 0.85, df = 1 (P = 0.36); I² = 0%

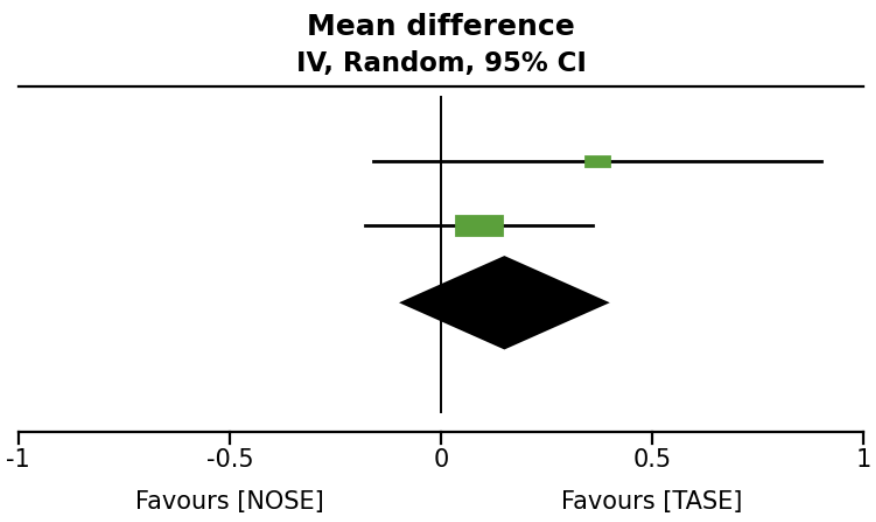

Footnotes

a CI calculated by Wald-type method.

b Tau² calculated by Restricted Maximum-Likelihood method.

Supplementary Figure S2. Leave-one-out sensitivity analysis for the pooled PFDI-20 score. Forest plots show the pooled mean difference in PFDI-20 score between transanal NOSE and TASE for (a) all four contributing studies and (b–e) after sequential omission of each study. Pooled estimates were calculated using a random-effects model. The pooled estimate remained small and statistically non-significant throughout.

(a) All studies (main PFDI-20 analysis)

	NOSE			TASE				
**Study or Subgroup**	**Mean**	**SD**	**Total**	**Mean**	**SD**	**Total**	**Weight**	**MD 95% CI**
Ju 2023	7.1	1.4	91	7.4	1.3	91	25.1%	-0.30 [-0.69, 0.09]
Li 2022	6.56	1.36	106	6.47	1.87	106	20.0%	0.09 [-0.35, 0.53]
Tang 2021	7.01	1.37	186	6.95	1.5	186	45.2%	0.06 [-0.23, 0.35]
Zhu 2021	6.56	1.71	59	6.43	1.79	59	9.7%	0.13 [-0.50, 0.76]
**Total**			**442**			**442**	**100.0%**	**-0.02 [-0.21, 0.18]**

Test for overall effect: Z = 0.17 (P = 0.86)

Heterogeneity: Tau² (REML) = 0.00; Chi² = 2.70, df = 3 (P = 0.44); I² = 1%

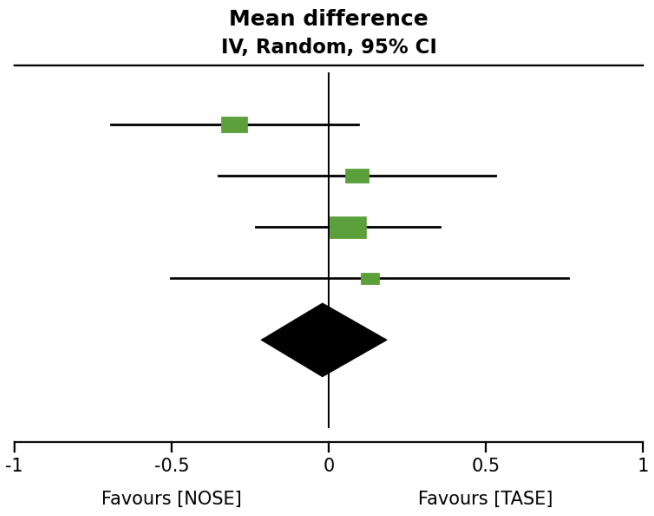

Footnotes

a CI calculated by Wald-type method.

b Tau² calculated by Restricted Maximum-Likelihood method.

(b) Omitting Tang 2021

	NOSE			TASE				
**Study or Subgroup**	**Mean**	**SD**	**Total**	**Mean**	**SD**	**Total**	**Weight**	**MD 95% CI**
Zhu 2021	6.56	1.71	59	6.43	1.79	59	20.0%	0.13 [-0.50, 0.76]
Li 2022	6.56	1.36	106	6.47	1.87	106	36.5%	0.09 [-0.35, 0.53]
Ju 2023	7.1	1.4	91	7.4	1.3	91	43.5%	-0.30 [-0.69, 0.09]
**Total**			**256**			**256**	**100.0%**	**-0.07 [-0.37, 0.23]**

Test for overall effect: Z = 0.47 (P = 0.64)

Heterogeneity: Tau² (REML) = 0.01; Chi² = 2.20, df = 2 (P = 0.33); I² = 9%

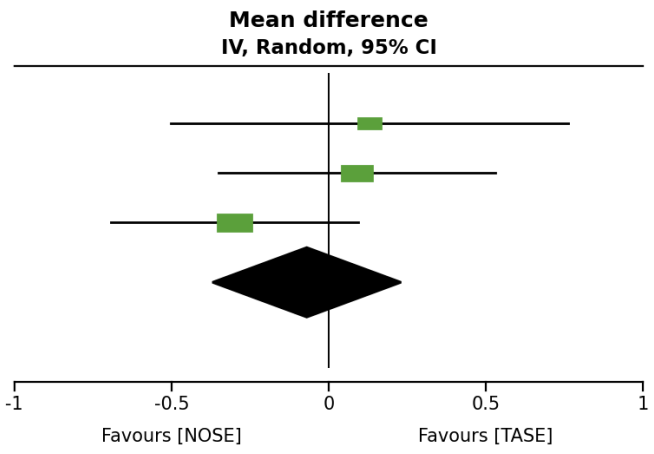

Footnotes

a CI calculated by Wald-type method.

b Tau² calculated by Restricted Maximum-Likelihood method.

(c) Omitting Zhu 2021

	NOSE			TASE				
**Study or Subgroup**	**Mean**	**SD**	**Total**	**Mean**	**SD**	**Total**	**Weight**	**MD 95% CI**
Ju 2023	7.1	1.4	91	7.4	1.3	91	29.2%	-0.30 [-0.69, 0.09]
Li 2022	6.56	1.36	106	6.47	1.87	106	24.0%	0.09 [-0.35, 0.53]
Tang 2021	7.01	1.37	186	6.95	1.5	186	46.8%	0.06 [-0.23, 0.35]
**Total**			**383**			**383**	**100.0%**	**-0.04 [-0.27, 0.19]**

Test for overall effect: Z = 0.32 (P = 0.75)

Heterogeneity: Tau² (REML) = 0.01; Chi² = 2.47, df = 2 (P = 0.29); I² = 18%

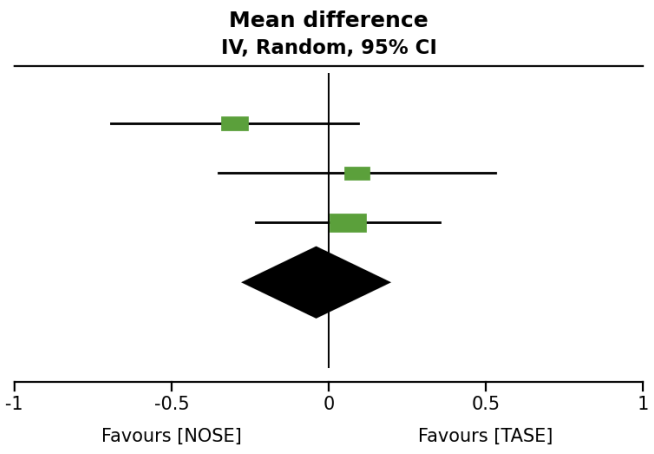

Footnotes

a CI calculated by Wald-type method.

b Tau² calculated by Restricted Maximum-Likelihood method.

(d) Omitting Li 2022

	NOSE			TASE				
**Study or Subgroup**	**Mean**	**SD**	**Total**	**Mean**	**SD**	**Total**	**Weight**	**MD 95% CI**
Ju 2023	7.1	1.4	91	7.4	1.3	91	33.8%	-0.30 [-0.69, 0.09]
Tang 2021	7.01	1.37	186	6.95	1.5	186	50.7%	0.06 [-0.23, 0.35]
Zhu 2021	6.56	1.71	59	6.43	1.79	59	15.5%	0.13 [-0.50, 0.76]
**Total**			**336**			**336**	**100.0%**	**-0.05 [-0.32, 0.21]**

Test for overall effect: Z = 0.38 (P = 0.71)

Heterogeneity: Tau² (REML) = 0.01; Chi² = 2.41, df = 2 (P = 0.30); I² = 24%

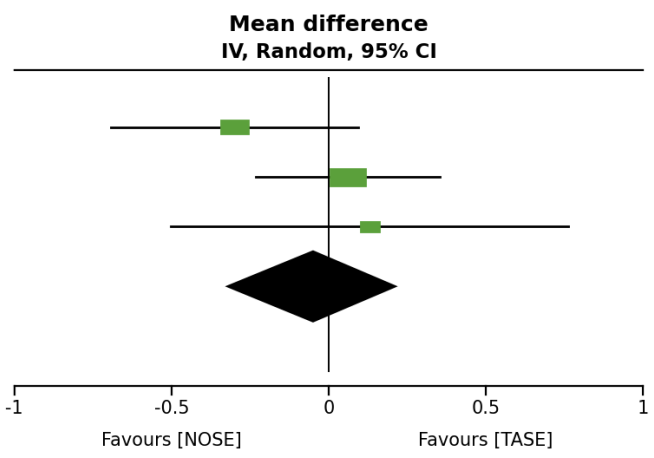

Footnotes

a CI calculated by Wald-type method.

b Tau² calculated by Restricted Maximum-Likelihood method.

(e) Omitting Ju 2023

	NOSE			TASE				
**Study or Subgroup**	**Mean**	**SD**	**Total**	**Mean**	**SD**	**Total**	**Weight**	**MD 95% CI**
Li 2022	6.56	1.36	106	6.47	1.87	106	26.6%	0.09 [-0.35, 0.53]
Tang 2021	7.01	1.37	186	6.95	1.5	186	60.5%	0.06 [-0.23, 0.35]
Zhu 2021	6.56	1.71	59	6.43	1.79	59	12.9%	0.13 [-0.50, 0.76]
**Total**			**351**			**351**	**100.0%**	**0.08 [-0.15, 0.30]**

Test for overall effect: Z = 0.66 (P = 0.51)

Heterogeneity: Tau² (REML) = 0.00; Chi² = 0.04, df = 2 (P = 0.98); I² = 0%

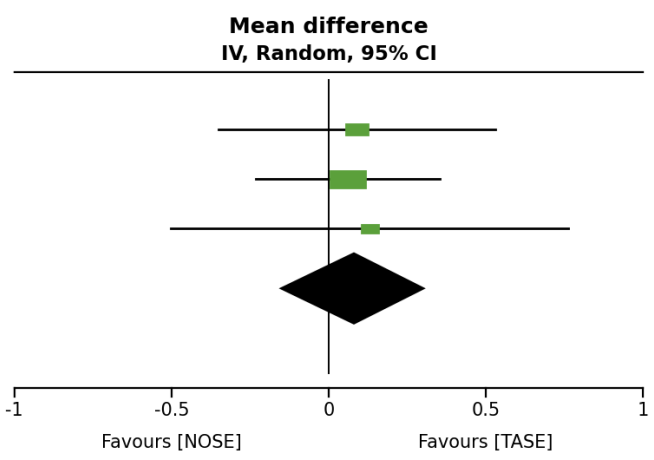

Footnotes

a CI calculated by Wald-type method.

b Tau² calculated by Restricted Maximum-Likelihood method.

Supplementary Figure S3. Overlap-adjusted pooled analysis excluding potentially overlapping Affiliated Hospital of Harbin Medical University cohorts. (a) Wexner incontinence score and (b) PFDI-20 score, each pooled using a fixed-effect model after retaining a single representative Harbin cohort per outcome and excluding the remaining potentially overlapping Harbin studies. Estimates remained consistent in direction and magnitude with the primary analyses, with reduced precision.

(a) Wexner incontinence score — overlap-adjusted (fixed-effect)

	NOSE			TASE				
**Study or Subgroup**	**Mean**	**SD**	**Total**	**Mean**	**SD**	**Total**	**Weight**	**MD 95% CI**
Xu 2024	3.5	1.2	132	3.3	1.1	132	78.5%	0.20 [-0.08, 0.48]
Zhang J 2022	1.28	1.62	69	0.91	1.56	69	21.5%	0.37 [-0.16, 0.90]
**Total**			**201**			**201**	**100.0%**	**0.24 [-0.01, 0.48]**

Test for overall effect: Z = 1.88 (P = 0.06)

Heterogeneity: Chi² = 0.31, df = 1 (P = 0.58); I² = 0%

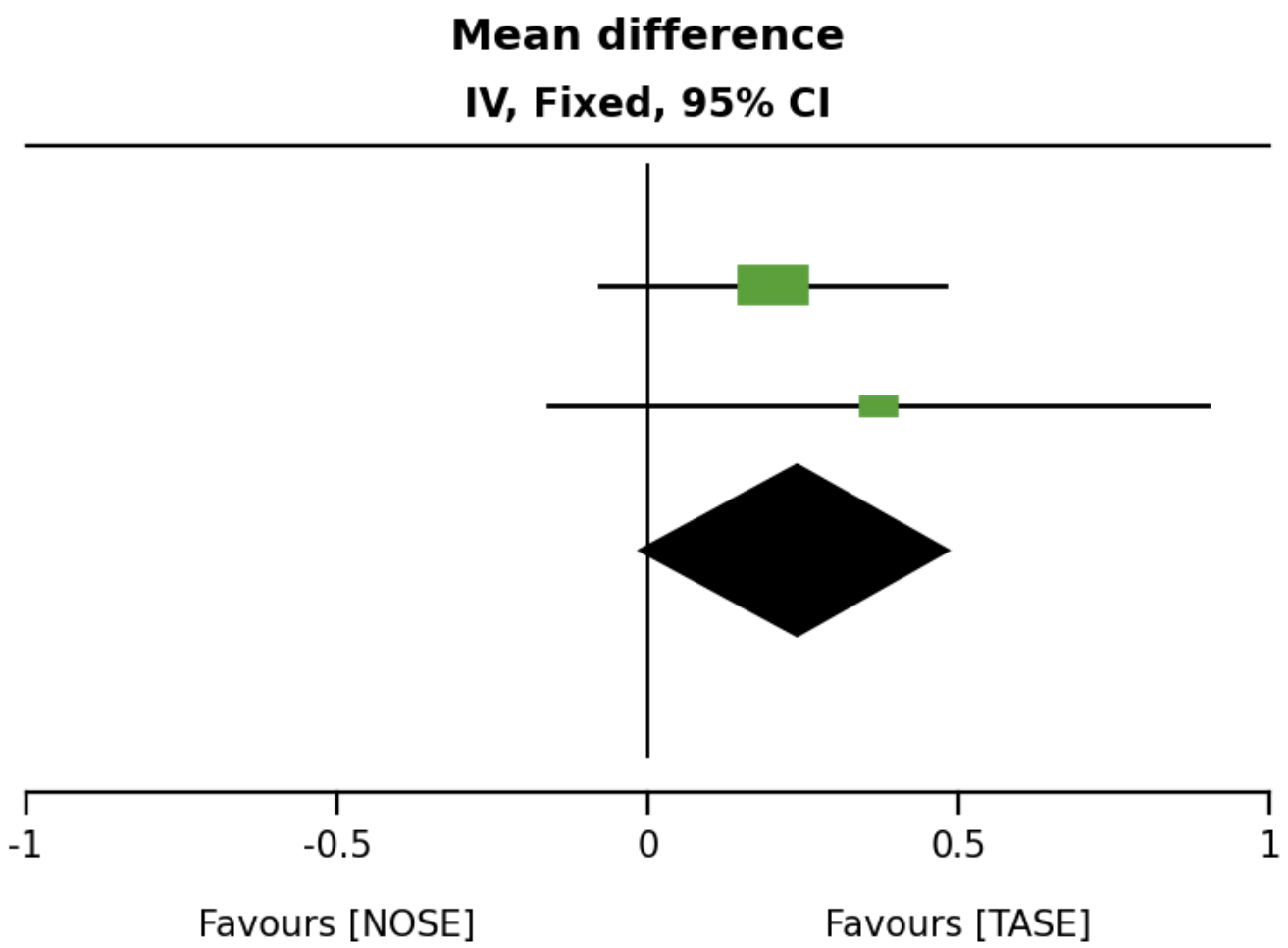

(b) PFDI-20 score — overlap-adjusted (fixed-effect)

	NOSE			TASE				
**Study or Subgroup**	**Mean**	**SD**	**Total**	**Mean**	**SD**	**Total**	**Weight**	**MD 95% CI**
Ju 2023	7.1	1.4	91	7.4	1.3	91	55.7%	-0.30 [-0.69, 0.09]
Li 2022	6.56	1.36	106	6.47	1.87	106	44.3%	0.09 [-0.35, 0.53]
**Total**			**197**			**197**	**100.0%**	**-0.13 [-0.42, 0.17]**

Test for overall effect: Z = 0.85 (P = 0.39)

Heterogeneity: Chi² = 1.68, df = 1 (P = 0.19); I² = 40%

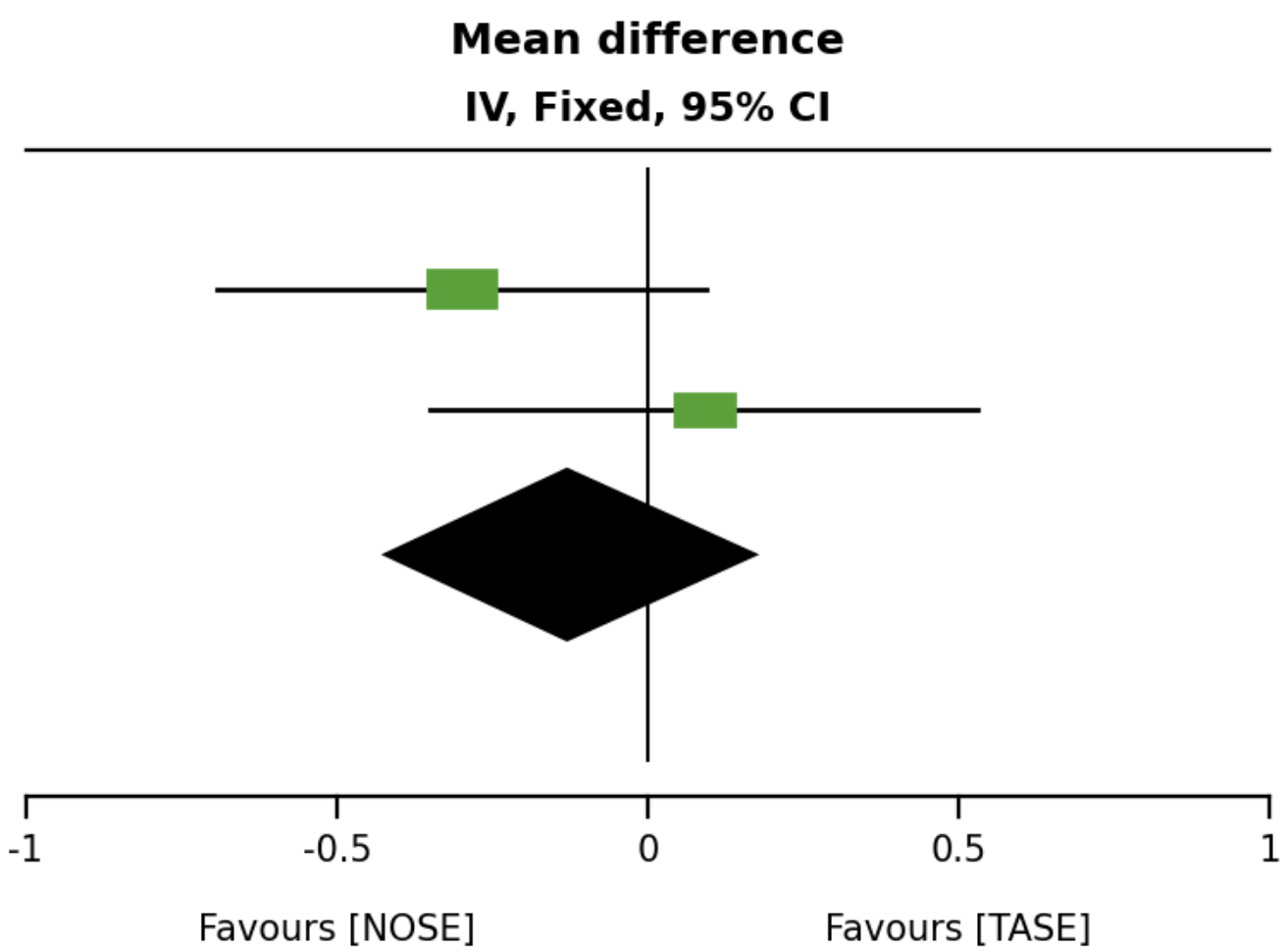

Supplement: Supplementary file 1 [file 423_2026_4184_MOESM1_ESM.pdf]
